# The Association Between Congestive Heart Failure and One-Year Mortality After Surgery in Singaporean Adults: A Secondary Retrospective Cohort Study Using Propensity-Score Matching, Propensity Adjustment, and Propensity-Based Weighting

**DOI:** 10.3389/fcvm.2022.858068

**Published:** 2022-06-17

**Authors:** Yong Han, Haofei Hu, Yufei Liu, Qiming Li, Zhiqiang Huang, Zhibin Wang, Dehong Liu, Longning Wei

**Affiliations:** ^1^Department of Emergency, Shenzhen Second People’s Hospital, Shenzhen, China; ^2^Department of Nephrology, Shenzhen Second People’s Hospital, Shenzhen, China; ^3^Department of Neurosurgery, Shenzhen Second People’s Hospital, Shenzhen, China; ^4^Department of Emergency, Hechi People’s Hospital, Hechi, China

**Keywords:** heart failure, standardised mortality ratio-weighted, propensity-score matching, inverse-probability-of-treatment-weighted, mortality

## Abstract

**Background:**

Although congestive heart failure (CHF) is considered a risk factor for postoperative mortality, reliable quantification of the relationship between CHF and postoperative mortality risk is limited. We aimed to investigate the association between CHF and 1-year mortality after surgery in a large cohort of the Singaporean population.

**Methods:**

In this retrospective cohort study, the study population included 69,032 adult patients who underwent surgery at Singapore General Hospital between 1 January 2012 and 31 October 2016. The target independent and dependent variables were CHF and 1-year mortality after surgery, respectively. Propensity score was estimated using a non-parsimonious multivariable logistic regression model. Multivariable adjustment, propensity score matching, propensity score adjustment, and propensity score-based weighting Cox proportional-hazards regression were performed to investigate the association between CHF and 1-year mortality after surgery.

**Results:**

The multivariate-adjusted hazard ratio (HR) in the original cohort was 1.39 (95% confidence interval (CI): 1.20–1.61, *P* < 0.001). In additional propensity score adjustment, the HR between CHF and 1-year mortality after surgery was 1.34 (95% CI: 1.15–1.56, *P* < 0.001). In the propensity score-matched cohort, the multivariate-adjusted Cox proportional hazard regression model analysis showed participants with CHF had a 54% increased risk of 1-year mortality after surgery (HR 1.54, 95% CI: 1.19–1.98, *P* < 0.001). The multivariate-adjusted HR of the inverse probability of treatment-weighted and standardised mortality ratio-weighted cohorts was 1.34 (95% CI: 1.10–1.62, *P* = 0.004) and 1.24 (95% CI: 1.17–1.32, *P* < 0.001), respectively.

**Conclusion:**

CHF is an independent risk factor for 1-year mortality after surgery in patients undergoing surgery. Depending on the statistical method, patients with CHF had a 24–54% increased risk of 1-year all-cause mortality after surgery. This provides a reference for optimising clinical decision-making, improving preoperative consultation, and promoting clinical communication.

## Introduction

Congestive heart failure (CHF) is the leading cause of morbidity and mortality worldwide (1). Its prevalence is more than 22 million cases worldwide, with the incidence of 2 million new cases per year (2). The 5-year mortality rate is 62% for women and 75% for men after CHF (3). With advancements in medical care, an increasing number of patients with CHF may select surgery to treat certain diseases. Therefore, it is imperative to improve our understanding of the impact of HF on postoperative outcomes. Although CHF has been recognised as a risk factor for postoperative mortality and has been incorporated into several risk indicators (4–8), few studies have examined the relationship between CHF and postoperative mortality. The hazard ratio (HR)/odds ratio (OR) produced by previous studies on the relationship between CHF and the prognosis of surgical patients varied widely (9–11).

Previous studies have mainly applied traditional parsimonious regression models based on analytical adjustments to control for confounders. However, such models may still result in a bias owing to unmeasured or residual confounding of the model and overfitting (12, 13). Research methods based on propensity score (PS) are considered the core alternative for controlling the confounding of observational research. Both large and small sample theories show that adjustment for the scalar PS is sufficient to remove bias due to all observed covariates (14, 15). Several adjustment methods incorporating the estimated PS have been proposed, including matching, regression adjustment, and weighting (13, 15–18).

Therefore, based on the current status of research on the impact of CHF on the prognosis of surgical patients, no studies have applied PS-based methods to investigate this relationship. We used a large sample of patients discharged from a general hospital in Singapore to study the differential impact of CHF on surgical outcomes using various statistical models.

## Materials and Methods

### Data Source and Participants

This was a post-mortem analysis of a large vertical cohort established by Yilin Eileen Sim’s team in Singapore. The analysed data were stored in the Dryad database^1^ by Yilin et al. The author of the original study waived all copyrights and related ownership of these data. Therefore, we can use these data for secondary analysis without infringing on the rights of the author. The data were obtained from a published paper: Sim et al. (19).

These clinical records were obtained from the hospital’s clinical information system [Sunrise Clinical Manager (SCM); Allscripts, IL, United States]. The mortality and follow-up data of the original study were paired with their national electronic health records. The variables included in the database file were as follows: sex, age, race, history of previous cerebrovascular accidents (CVA), history of ischaemic heart disease (IHD), history of diabetes mellitus on insulin (DMI), history of CHF, creatinine category, priority of surgery, surgical risk classification, American Society of Anaesthesiologists Physical Status (ASA-PS), red cell distribution width (RDW) category, stage of chronic kidney disease (CKD), degree of anaemia, type of anaesthesia (general or regional anaesthesia), follow-up days, and survival status (19, 20). The original study was approved by the Institutional Review Board (SingHealth CIRB 2014/651/D), which waived the requirement for individual informed consent (19). As reported elsewhere, the current study is a secondary analysis of the original study and did not receive ethical approval (21).

### Study Sample

**FIGURE 1:**
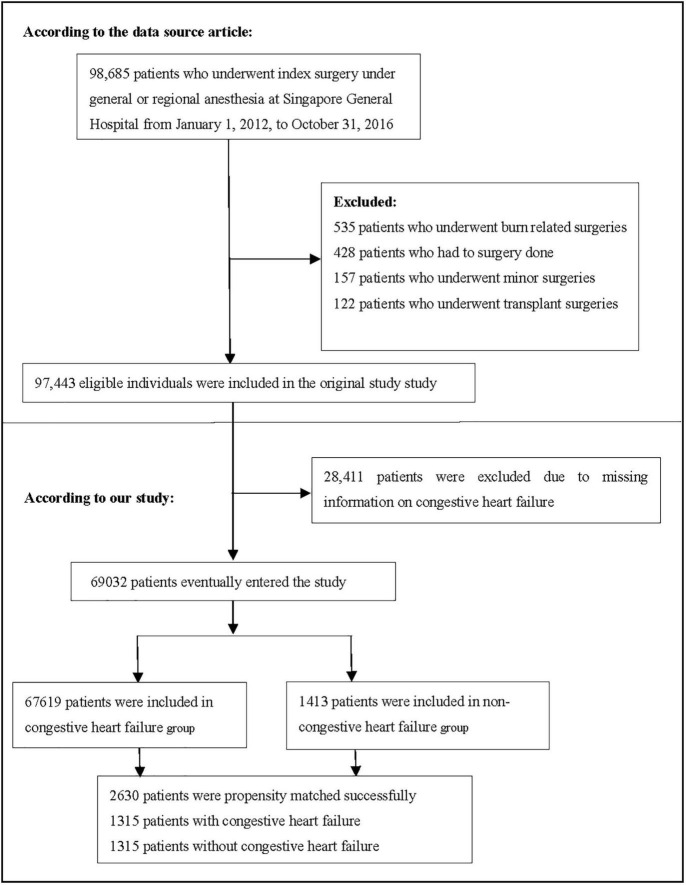
Flowchart of study participants. It showed the process of screening participants.

Consistent with the original study, patients who underwent surgery at Singapore General Hospital from 1 January 2012 to 31 October 2016 and were aged ≥18 years were included. The original research exclusion criteria were as follows: (1) patients who underwent transplantation and burn surgeries, (2) patients who had no surgery, and (3) patients who underwent minor surgeries (19). The final dataset consisted of 97,443 participants. In the present study, we excluded participants with missing CHF information (n = 28,411). Figure 1 depicts the participant selection process. Finally, our study included a total of 69,032 participants for secondary analysis.

### Variable Definitions

Laboratory examinations were performed within 90 days of surgery. These included serum creatinine level, serum haemoglobin, and RDW. Anaemia was defined by the World Health Organization gender-based classification of anaemia severity (19, 22). The severity of anaemia is characterised as follows: mild anaemia (haemoglobin concentration: male 11–12.9 g/dL, female 11–11.9 g/dL); moderate anaemia (haemoglobin concentration: 8.0–10.9 g/dL), and severe anaemia (haemoglobin concentration: 8.0 g/dL). RDW was reported as a coefficient of variation (percentage) of red blood cell volume; levels above 15.7% were defined as high RDW, and the normal reference range for RDW was 10.9–15.7%. The target independent variable was the presence or absence of CHF history obtained at baseline. The dependent variable was 1-year mortality event during follow-up. Of the study patients, 88.7% were followed up for 1 year and mean duration of follow-up was 258 days (19).

### Missing Data Processing

Missing data in observational studies are a frequently encountered problem that cannot be fully avoided. Missing data accounted for 2.71% of all variables in the dataset analysed in this study (Supplementary Table 1). To reduce the deviation caused by missing covariates, which cannot reflect the statistical efficiency of the target sample in the modelling process, missing data in this study adopted multiple imputations (23, 24). In the present study, the analysis steps in the original and imputed datasets were calculated and compared. Similar core results were obtained using the original and imputed datasets; thus, we report the results for the imputed dataset (Supplementary Table 2).

### Statistical Analysis

Participants were stratified by CHF, continuous variables are expressed as median (quartile) (skewed distribution) or mean ± standard deviation (normal distribution), and categorical variables are expressed as a frequency or percentage. Two-sample *t*-tests were performed for normally distributed continuous variables, Wilcoxon rank-sum tests for non-normally distributed continuous variables and ordered categorical variables, and chi-square tests for categorical variables.

The study goal was to use different methods to control for confounding factors and evaluate the impact of CHF on the 1-year postoperative mortality of patients who underwent surgery. Specifically, we applied five methods: the original cohort multivariate-adjusted Cox proportional-hazards regression model and four PS methods. The four PS methods included PS matching multivariate-adjusted Cox proportional-hazards regression model; PS adjustment Cox proportional-hazards regression model; inverse probability of treatment-weighted (IPTW) model, and standardised mortality ratio (SMR)-weighted multivariate-adjusted Cox proportional-hazards regression model. (1) PS was estimated using a non-parsimonious multivariable logistic regression model wherein CHF was used as the independent variable, and all baseline characteristics listed in Table 1 were covariates (25). The variables used for matching included age, sex, race, CVA, IHD, DMI, stage of CKD, degree of anaemia, priority of surgery and surgical risk classification, ASA-PS, RDW category, type of anaesthesia, matching using a 1:1 matching protocol, no replacement (greedy matching algorithm), and calliper width of 0.0005. Standardised differences (SDs) were estimated for all baseline covariates before and after matching to assess pre-matched and post-matched balance. SD of <20.0% for a given covariate indicate a relatively small imbalance (26–28). We used a multivariate-adjusted Cox proportional hazard regression model to evaluate the association between CHF and 1-year mortality after surgery in the PS-matched cohort. (2) PS adjustment: a multivariable Cox proportional-hazards model with the same strata and covariates, with additional adjustment for PS. The analysis included all patients (29–31). (3) The IPTW estimator estimates the treatment effect in a population whose risk factor distribution is equal to that found in all study subjects. IPTW was calculated as the inverse of the PS of patients with combined CHF and the inverse of (1 - PS) for non-CHF patients. The IPTW model was applied to generate a weighted cohort (15, 31, 32). IPTW multivariate-adjusted Cox proportional-hazards regression model has the same strata and covariates with inverse probability weighting according to PS. The analysis included all patients (31). (4) The SMR-weighted estimator estimates the treatment effect in a population whose distribution of risk factors is equal to that found in the treated study subjects only. SMR-weighted analyses used 1 as the value of the CHF group and the probability of propensity PS/(1 - PS) for the non-CHF group as weights and estimated the standardised effect measure for the CHF group (exposed group) as the standard population (15, 18). SMR-weighted multivariate-adjusted Cox proportional-hazards regression model, with the same strata and covariates with inverse probability weighting according to PS. All patients were included in the analysis. Cumulative mortality was used to describe mortality (15, 33). When added to the model, the covariates that changed the hazard ratios by at least 10% were considered confounders and were adjusted for in the multivariate analysis (34). In addition, Kaplan–Meier analysis and log-rank tests were performed to compare 1-year mortality after surgery.

**TABLE 1:** Baseline characteristics before and after propensity score matching.

	Before matching			After matching		
	CHF	Non-CHF	SD (100%)	CHF	Non-CHF	SD (100%)
Participants	1,413	67,619		1,315	1,315	
Age(years)			80.5			115.3
18–<30	7 (0.495%)	7,281 (10.768%)		6 (0.456%)	1 (0.076%)	
30–49	127 (8.988%)	19,047 (28.168%)		121 (9.202%)	645 (49.049%)	
50–69	737 (52.159%)	29,532 (43.674%)		686 (52.167%)	578 (43.954%)	
≥70	542 (38.358%)	11,759 (17.390%)		502 (38.175%)	91 (6.920%)	
Sex			28.3			1.4
Female	537 (38.004%)	35,139 (51.966%)		508 (38.631%)	517 (39.316%)	
Male	876 (61.996%)	32,480 (48.034%)		807 (61.369%)	798 (60.684%)	
Race			26.8			25.0
Chinese	975 (69.002%)	48,641 (71.934%)		923 (70.190%)	796 (60.532%)	
Malay	227 (16.065%)	6,641 (9.821%)		210 (15.970%)	219 (16.654%)	
Indian	147 (10.403%)	5,869 (8.680%)		126 (9.582%)	188 (14.297%)	
Others	64 (4.529%)	6,468 (9.565%)		56 (4.259%)	112 (8.517%)	
ASA-PS			220.5			20.0
1	4 (0.283%)	16,216 (23.981%)		4 (0.304%)	2 (0.152%)	
2	103 (7.289%)	38,212 (56.511%)		103 (7.833%)	68 (5.171%)	
3	1023 (72.399%)	12,122 (17.927%)		1010 (76.806%)	970 (73.764%)	
4	279 (19.745%)	1,046 (1.547%)		198 (15.057%)	268 (20.380%)	
5	4 (0.283%)	23 (0.034%)		0 (0.000%)	7 (0.532%)	
CVA			38.5			3.2
No	1237 (87.544%)	65,918 (97.484%)		1165 (88.593%)	1178 (89.582%)	
Yes	176 (12.456%)	1,701 (2.516%)		150 (11.407%)	137 (10.418%)	
IHD			150.8			7.3
No	461 (32.626%)	61,604 (91.105%)		461 (35.057%)	507 (38.555%)	
Yes	952 (67.374%)	6,015 (8.895%)		854 (64.943%)	808 (61.445%)	
DMI			46.5			11.3
No	1179 (83.439%)	65,540 (96.925%)		1117 (84.943%)	1061 (80.684%)	
Yes	234 (16.561%)	2,079 (3.075%)		198 (15.057%)	254 (19.316%)	
Creatinine category			56.1			8.8
Normal	1132 (80.113%)	65,752 (97.239%)		1077 (81.901%)	1031 (78.403%)	
High	281 (19.887%)	1,867 (2.761%)		238 (18.099%)	284 (21.597%)	
Anemia			69.8			8.9
Normal	582 (41.189%)	49,564 (73.299%)		571 (43.422%)	546 (41.521%)	
Mild	380 (26.893%)	10,087 (14.917%)		356 (27.072%)	336 (25.551%)	
Moderate	432 (30.573%)	7,710 (11.402%)		370 (28.137%)	404 (30.722%)	
Severe	19 (1.345%)	258 (0.382%)		18 (1.369%)	29 (2.205%)	
Stage of CKD			100.7			29.5
1	326 (23.071%)	41,181 (60.902%)		316 (24.030%)	383 (29.125%)	
2	442 (31.281%)	19,798 (29.279%)		424 (32.243%)	325 (24.715%)	
3	358 (25.336%)	4,316 (6.383%)		329 (25.019%)	242 (18.403%)	
4–5	287 (20.311%)	2,324 (3.437%)		246 (18.707%)	365 (27.757%)	
Anaesthesia			18.3			14.5
General	1114 (78.839%)	58,019 (85.803%)		1032 (78.479%)	1106 (84.106%)	
Regional	299 (21.161%)	9,600 (14.197%)		283 (21.521%)	209 (15.894%)	
Priority of surgery			8.1			3.2
Elective	1123 (79.476%)	55,884 (82.645%)		1050 (79.848%)	1033 (78.555%)	
Emergency	290 (20.524%)	11,735 (17.355%)		265 (20.152%)	282 (21.445%)	
Surgery risk			31.0			1.4
Low	556 (39.349%)	33,912 (50.152%)		521 (39.620%)	518 (39.392%)	
Moderate	663 (46.921%)	29,841 (44.131%)		626 (47.605%)	623 (47.376%)	
High	194 (13.730%)	3,866 (5.717%)		168 (12.776%)	174 (13.232%)	
RDW category			38.2			14.0
≤15.7%	1084 (76.716%)	61,247 (90.577%)		1034 (78.631%)	955 (72.624%)	
>15.7%	329 (23.284%)	6,372 (9.423%)		281 (21.369%)	360 (27.376%)	

*Values were n (%) or mean ± SD.*

*SD, standardised differences; CVA, history of previous cerebrovascular accidents; IHD, history of ischemic heart disease; DMI, history of diabetes mellitus on insulin; CHF, congestive heart failure; ASA-PS, American society of anaesthesiologists physical Status; CKD, chronic kidney disease; RDW, red cell distribution width.*

We performed a series of sensitivity analyses to test the robustness of the results; first, because of the extreme difference in the mortality OR among the low- and high-propensity strata. Mortality was lower in patients with low PS. Therefore, we excluded participants with a PS of <0.05 and performed a sensitivity analysis using five models. Second, bias may arise because substantial CHF information was missing. We applied multiple imputations to estimate missing CHF values (n = 28,411). The imputation model included age, sex, race, CHF, CVA, IHD, DMI, stage of CKD, degree of anaemia, priority of surgery and surgical risk classification, ASA-PS, RDW category, and type of anaesthesia. Multiple statistical models were used to analyse the association between CHF and postoperative mortality in patients to verify the reliability of our analysis after excluding participants with missing CHF information. In addition, we explored the possibility of unmeasurable confounding between CHF and 1-year mortality after surgery by calculating E-values (35).

Differences in age and CKD stage remained between the CHF and non-CHF groups after PS matching. This may be associated with increased mortality in surgical patients (36). This may lead to overestimation of the relationship between CHF and 1-year mortality after surgery. Therefore, we stratified the participants according to CKD (stages 1–2 vs. 3–5) (37, 38). Pre-specified subgroup analyses were performed based on these two characteristics. The subgroups were based on age and CKD stage. Each stratification adjusted for all factors except the stratification factor itself. Only the corresponding matched pairs in the same subgroup were selected to maintain the balance of baseline characteristics between the CHF and non-CHF groups in subgroup analyses.

All results are reported according to the STROBE statement (39). All analyses were performed using the statistical software packages R^2^ (The R Foundation) and Empower-Stats^3^ (X&Y Solutions, Inc., Boston, MA, United States). All tests were two-tailed, and *P*-values of <0.05 were regarded as statistically significant.

## Results

**FIGURE 2:**
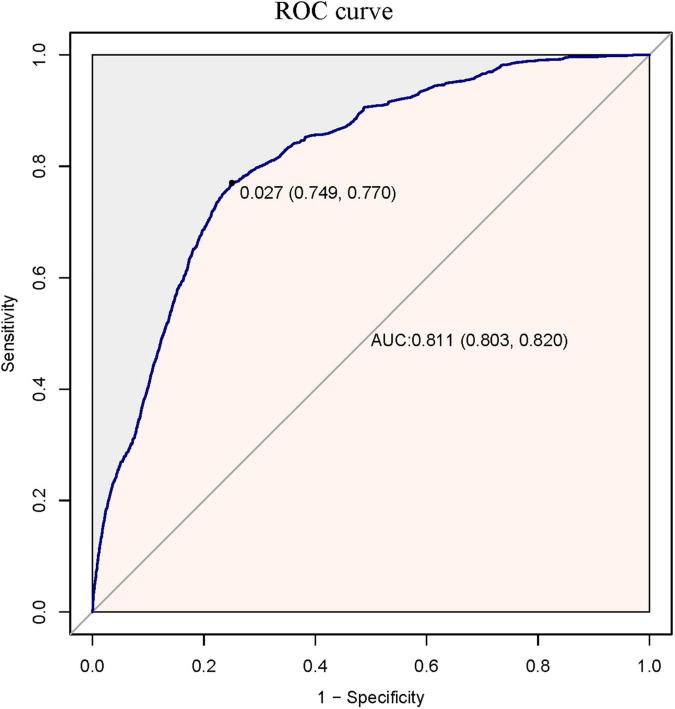
The ROC curve of propensity score to predict one-year mortality after surgery. It showed that the logistic model used to estimate the propensity score yielded a c-statistic of 0.811.

A total of 69,032 participants (48.32% men and 51.68% women) were included in the analysis. Among them, 1,413 (2.05%) had CHF and 67,619 (97.95%) did not have CHF. The number of participants aged 18–29, 30–49, 50–69, and ≥70 was 7,288 (10.562%), 19,174 (27.78%), 30,269 (43.85%) and 12,301 (17.82%), respectively. PS was estimated using a non-parsimonious multivariable logistic regression model, and CHF was used as the independent variable. The variables used for matching included age, sex, race, CVA, IHD, DMI, stage of CKD, degree of anaemia, priority of surgery and surgical risk classification, ASA-PS, RDW category, type of anaesthesia, matching using a 1:1 matching protocol, no replacement (greedy matching algorithm), and calliper width of 0.0005. Before PS matching, there were differences in almost all baseline characteristics between the CHF and non-CHF groups (Table 1). After one-to-one matching using PS analysis, 1,315 patients with CHF were successfully matched with 1,315 non-CHF participants. After matching, except race and age, the SD for almost all variables were <20.0%, indicating that the PSs were well matched. In other words, there was only a small difference in baseline characteristics between the CHF and non-CHF groups (Table 1). The logistic model used to estimate PS yielded a c-statistic of 0.811 (Figure 2).

**FIGURE 3:**
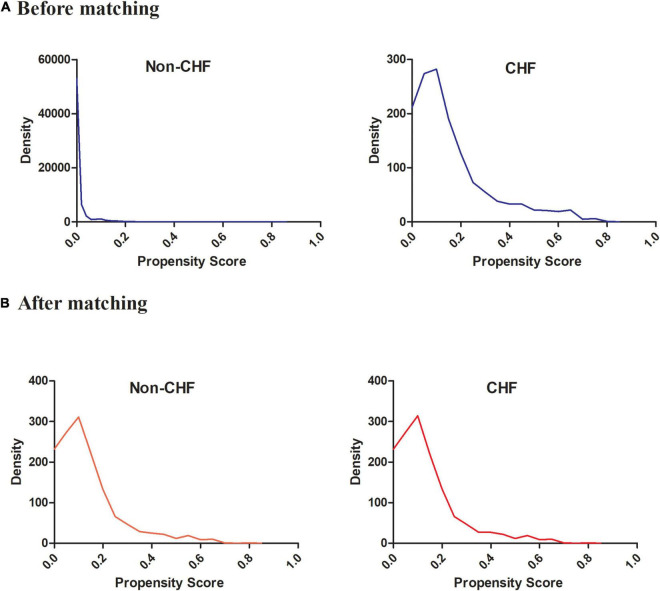
The probability density functions of the propensity score for CHF and non-CHF participants **(A)** before matching and **(B)** after matching. It showed the probability density functions of the propensity score for congestive heart failure and non-congestive heart failure participants before matching and after matching.

Supplementary Figure 1 showed that in the original cohort, the mean PS was 0.017 ± 0.049 for the CHF group and 0.163 ± 0.159 for the non-CHF group (*P* < 0.01). However, the mean PS in the matched population was 0.166 ± 0.125 for both groups (*P* = 0.995). (Supplementary Figure 2). The probability density functions of the PS for the CHF and non-CHF groups are summarised in Figure 3. As expected, the distribution of PS for the non-CHF group shifted toward 0 and for the CHF group shifted somewhat toward 1. The figure also illustrates that the overlap of PS for the CHF and non-CHF groups is limited to a narrow range (Figure 3A and Supplementary Figure 3A). The probability density functions of PS for the CHF and non-CHF groups after matching are summarised in Figure 3B. As expected, the distribution of PS for the CHF and non-CHF groups remained basically the same (Supplementary Figure 3B).

### Propensity Stratum-Specific Effects

For 1-year mortality after surgery, the gradients across levels of PS for the CHF and non-CHF groups were strong and unexpectedly different. Supplementary Table 3 summarises the proportions of patient mortality during the follow-up period in the CHF and non-CHF groups according to PS percentiles. We noted some important considerations. First, the PS of very few patients in the CHF group was below the 70th percentile of the overall PS. Second, the mortality rate of both groups increased as PS increased. The associated empirical OR for 1-year mortality after surgery increased from 0.821 in the 70th–80th percentiles of PS to 1.247 in the 99th percentile (Supplementary Table 3).

### One-Year Mortality After Surgery

**FIGURE 4:**
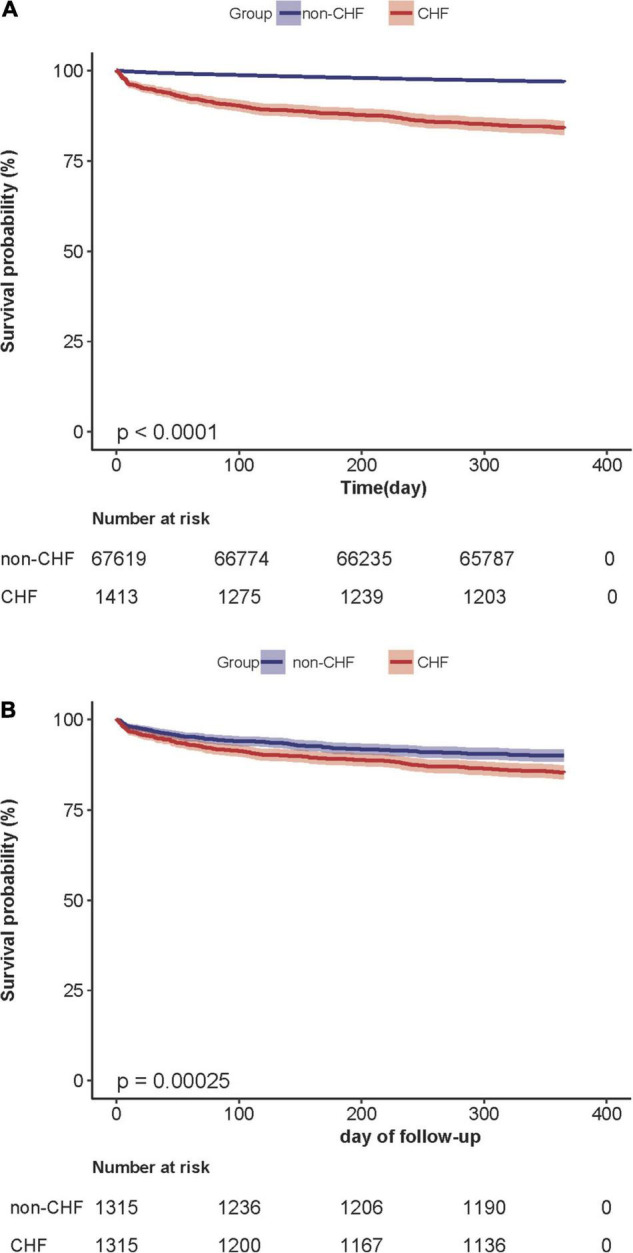
**(A)** Kaplan–Meier survival curve based on congestive heart failure in the original cohort. Kaplan–Meier analysis of one-year mortality after surgery based on congestive heart failure(CHF) and non-congestive heart failure (non-CHF) in the original cohort(log-rank, *P* < 0.0001). **(B)** Kaplan– Meier survival curve based on congestive heart failure in the propensity-score matching cohort. Kaplan–Meier analysis of one-year mortality after surgery based on congestive heart failure (CHF) and non-congestive heart failure (non-CHF) in the propensity-score matching cohort (log-rank, *P* = 0.0002).

Supplementary Table 4 showed the 1-year mortality after surgery of the CHF and non-CHF groups before and after PS matching. Before PS matching, 2,307 participants died during the follow-up period. The corresponding mortality rates in the CHF and non-CHF groups were 15.9% (95% confidence interval (CI): 14.0–17.8) and 3.1% (95% CI: 2.9–3.2), respectively. After PS matching, the difference in mortality between the two groups changed significantly; the corresponding mortality rates in the CHF and non-CHF groups were 14.8% (95% CI: 12.8–16.7) and 10.0% (95% CI: 8.4–11.7). Kaplan–Meier analysis showed that the CHF group had a higher 1-year mortality after surgery than the non-CHF group in the original cohort (*P* < 0.001). After PS matching, the difference in mortality between the two groups significantly reduced (Figure 4).

### Analysis Results Through Different Confounding Control Methods

We used the Cox proportional-hazards regression model to assess the association between CHF and 1-year mortality after surgery in the original, PS matching, and weighted cohorts. In the original cohort, CHF was significantly associated with 1-year mortality after surgery (HR, 5.65; 95% CI: 4.92–6.48, *P* < 0.001). In other words, compared with participants without CHF, those with CHF had a 4.65-fold increased risk of 1-year mortality after surgery. After multivariate adjustment (adjusted for sex, age, race, CVA, IHD, DMI, priority of surgery, surgical risk classification, ASA-PS, stage of CKD, degree of anaemia, and type of anaesthesia), the association still existed (HR: 1.39, 95% CI: 1.20–1.61, *P* < 0.001). In PS adjustment (adjusted variables include PSs and the same strata and covariates), the HR between CHF and 1-year mortality after surgery was 1.34 (95% CI: 1.15–1.56, *P* < 0.001). Second, in the PS-matched cohort, the multivariate-adjusted Cox proportional hazard regression model analysis showed that the HR between CHF and 1-year mortality after surgery was 1.54 (95% CI: 1.19–1.98, *P* < 0.001). The adjusted variables were the same as those used in the original cohort. Finally, in the weighted cohort, after multivariate adjustment, the SMR-weighted analysis yielded an HR of 1.34 (95% CI: 1.10–1.62, *P* < 0.001) and the result of the IPTW multivariate-adjusted Cox proportional-hazards regression model analysis showed that the HR between CHF and 1-year mortality after surgery was 1.24 (95% CI: 1.17–1.32, *P* < 0.001). It should be emphasised that in the original cohort multivariate adjustment, PS matching multivariate adjustment, IPTW, and SMR-weighted multivariate adjustment, the adjusted variables were the same, including sex, age, race, CVA, IHD, DMI, priority of surgery, surgical risk classification, ASA-PS, stage of CKD, degree of anaemia, and type of anaesthesia. In PS adjustment, the adjusted variables included other model-adjusted variables and PS (Table 2).

**TABLE 2:** Associations between CHF and one-year postoperative mortality of surgical patients in the crude analysis, multivariable analysis, and four propensity-score methods analyses.

Cox proportional-hazards regression model	Adjusted variables	No.	HR	95%CI	*P*-value
Crude		69,032	5.65	4.92, 6.48	<0.001
Multivariable-adjusted model	Multivariable^†^	69,032	1.39	1.20, 1.61	<0.001
Propensity score adjustment	Propensity score + Multivariable^†^	69,032	1.34	1.15, 1.56	<0.001
Propensity score matching	Multivariable^†^	2,630	1.54	1.20, 1.98	<0.001
IPTW	Multivariable^†^	69,032	1.24	1.17, 1.32	<0.001
SMR–weighted	Multivariate^†^	69,032	1.34	1.10, 1.62	0.004

*HR, hazard ratio; CI, confidence interval; IPTW, inverse-probability-of-treatment weighted; SMR, standardised mortality ratio.*

*Multivariable†: Adjusted for gender, age, race, history of previous cerebrovascular accidents, history of ischemic heart disease, diabetes mellitus on insulin, priority of surgery, surgical risk classification, American society of anaesthesiologists physical status, stage of CKD, degree of anaemia, type of Anesthesia.*

### Sensitivity Analysis

We considered a significant difference in the associated empirical OR for 1-year mortality after surgery between the participants with low and high PSs (Supplementary Table 3). We further analysed patients with PS of ≥0.05. The crude HR for the restricted population was 1.81 (95% CI: 1.55–2.12, *P* < 0.001); the HRs for the five different methods were similar. The multivariate-adjusted HR in the original cohort was 1.59 (95% CI: 1.35–1.89, *P* < 0.001); PS adjustment revealed that the HR between CHF and 1-year mortality after surgery was 1.54 (95% CI: 1.30–1.85, *P* < 0.001). In the PS-matched cohort, after multivariate adjustment, HR was 1.55 (95% CI: 1.19–2.02, *P* < 0.001); the multivariate-adjusted HR of the IPTW and SMR-weighted cohorts was 1.49 (95% CI: 1.36–1.67, *P* < 0.001) and 1.45 (95% CI: 1.18–1.78, *P* < 0.001), respectively (Supplementary Table 5).

In addition, bias may arise due to the excessive missing CHF information. We applied multiple imputations to estimate missing CHF values (n = 28,411). After imputation, there were 2,101 participants in the CHF group and 95,342 in the non-CHF group. We applied five statistical models to the analysis, which yielded similar results (Supplementary Table 6). The HRs of the original cohort-adjusted model, PS adjustment model, PS matching adjusted, IPTW model, and SMR-weighted model were 1.24, 1.26, 1.31, 1.16, and 1.31, respectively. Therefore, excluding participants with missing CHF information did not affect the core findings of this study and suggests that our results are robust.

Furthermore, we generated an E-value to assess the sensitivity to unmeasured confounding factors. The E-value (2.13) was lower than the relative risk (3.34) of unmeasured confounders and 1-year mortality after surgery, suggesting that unmeasured or unknown confounders had little effect on the relationship between CHF and 1-year mortality after surgery. Our primary findings were robust.

### Subgroup Analysis

We performed subgroup analysis to examine the impact of potential confounders that may influence the association between CHF and 1-year mortality after surgery. Age and CKD stage were used as stratified variables to assess the trend of effect size. We observed interactions among the subgroups according to our specification. We found that age and CKD stage did not affect the relationship between 1-year mortality after surgery (all P-values for interaction < 0.05), respectively (Supplementary Table 7).

## Discussion

This one-to-one PS-matched cohort study showed that CHF was associated with a higher risk of mortality 1 year after surgery. After PS matching, CHF had a significant association with 1-year mortality after surgery, and the risk of mortality increased by 54% in the population with CHF (HR = 1.54, 95% CI: 1.1–1.98, *P* < 0.001). In PS adjustment, the HR between CHF and 1-year mortality after surgery was 1.34 (95% CI: 1.15–1.58, *P* < 0.001). We applied a Cox proportional-hazards model based on PS-based weighting to further verify the association between CHF and 1-year mortality after surgery. In the IPTW and SMR-weighted cohorts, compared with participants without CHF, those with CHF had a 24% and 34% increase in the risk of mortality 1 year after surgery, respectively.

We found that different methods of controlling confounding factors resulted in different HRs. The results of the PS matching multivariate-adjusted Cox proportional-hazards regression model were slightly higher, whereas the original cohort multivariate-adjusted, PS-adjusted, and SMR-weighted multivariate-adjusted Cox proportional-hazards regression models had similar hazard ratios. In comparison, the results of the IPTW multivariate-adjusted Cox proportional-hazards regression model were lower (Table 2). The number of participants without CHF was many times greater than the number of those with CHF. PS matching usually results in a successful match for almost all patients with CHF; many unmatched patients without CHF were excluded from the analysis. As a result, the distribution of covariates in the (successfully) matched subpopulation would be close to that in the treated study population. Most patients in the CHF group were in the propensity strata with a high risk of associated mortality, and the SMR-weighted method estimated the average effect of CHF in a population whose distribution of risk factors was equal to that found in the CHF group. Thus, it was not surprising that the SMR-weighted estimate was closer to the PS-matched estimate.

In contrast, the IPTW model estimated the average CHF effect for the entire study population. Given that 90% of the study population was in the propensity strata associated with low empirical odds ratios, the results of the IPTW multivariate-adjusted Cox proportional-hazards regression model were lower. The IPTW estimate increased to 1.49 when the patients in these three strata were excluded by restricting the analysis to the subpopulation of the CHF and non-CHF groups whose PS was ≥0.05. Indeed, once we restricted the analysis to subjects with PS of ≥0.05, all adjustment methods provided approximate results, with HR fluctuating around 1.50. The results of all these methods showed that the four PS methods could control for confounding factors well.

CHF is an established risk factor for poor prognosis after surgery across a wide range of specialties. A study conducted in Sweden showed that the crude and adjusted HRs for 30-day mortality after elective surgery in patients with CHF were 5.36 and 1.79, respectively (9). An analysis of 21,560,996 surgical hospitalisations revealed that the adjusted OR for in-hospital perioperative mortality in patients was 2.15 (11). Another study found similar results; compared with patients without CHF, patients with CHF had a 96% increased risk of 30-day mortality (10). Our study complements the existing literature supporting the hypothesis that CHF increases the risk of postoperative mortality in patients undergoing surgery. However, the estimated HR for the relationship between CHF and postoperative mortality was lower than that reported in previous studies.

We analysed these inconsistent findings, which may be justified by the following possible explanations: (1) The research population was different, including age, sex and race. As the original data failed to define the surgical category, our study population included all cardiac and non-cardiac surgery patients. Other studies have generally analysed these two populations separately. (2) Sample sizes in these studies varied widely. (3) The studies were adjusted for different covariates. (4) Previous studies mainly used variable adjustments to control confounding factors; this traditional parsimonious regression model could result in a bias because of unmeasured or residual confounding or overfitting of the model (40). We used PS methods to control for confounding factors and verify the association between CHF and 1-year mortality after surgery. (5) The outcome variables were different. Previous studies have focused on in-hospital or 30-day mortality rates. However, the dependent variable in our study was 1-year mortality after surgery.

Although the exact mechanism underlying the increased risk of postoperative death associated with CHF is unclear, the incidence of complications in this population may provide clues. A study found that, compared with patients without CHF, the incidence of crude complications in patients with CHF doubled (41). There is convincing evidence that this may be because patients with CHF have poor recovery, and even minor postoperative complications can significantly affect postoperative mortality (42).

Our study has two other strengths: (1) Our sample size was relatively larger than that of previous similar studies. (2) To the best of our knowledge, few cohort studies have used PS matching to explore the relationship between CHF and 1-year mortality after surgery. Research methods based on PSs are considered a core alternative for controlling the confounding effects of observational research.

The potential limitations of this study are as follows. First, the population included in this study was Singaporean and the race was mainly Chinese. Therefore, the universality of these results in other races requires further verification. Second, this study is a secondary analysis based on published data, so variables not included in the dataset cannot be adjusted. However, we used the E-value to evaluate the unmeasured confounding factors and found that our study was stable and reliable. Third, this study was based on a secondary analysis of published data and lacked some relevant information, such as recent surgical observations (perioperative and 30-day mortality), minor surgeries, and types of transplants. Fourth, PS methods balance the known confounding variables as much as possible. However, it could not ensure that all measured baseline characteristics were matched, and the influence of unknown variables was considered. However, we reduced the calliper width to 0.0005 to minimise the interference of some variables in the results. Fifth, differences in CKD stage and age remained between the CHF and non-CHF groups after PS matching. However, multivariate adjustment and subgroup analyses were performed. These analyses suggest that our results are robust. In addition, this observational study provides inferences about the association between CHF and 1-year mortality after surgery but cannot establish a causal relationship. Therefore, our findings need to be further validated by future prospective studies.

## Conclusion

CHF is an independent risk factor for 1-year mortality after surgery in patients undergoing surgery. This study quantified the relationship between CHF and surgical patient outcomes by applying various statistical models and presented a range of HR (1.24–1.54). This provides a reference for optimising clinical decision-making, improving preoperative consultation, and promoting clinical communication.

## Data Availability Statement

The datasets presented in this study can be found in online repositories. The names of the repository/repositories and accession number(s) can be found in the article/Supplementary Material.

## Ethics Statement

The studies involving human participants were reviewed and approved by Institutional Review Board (Singhealth CIRB 2014/651/D). Written informed consent for participation was not required for this study in accordance with the national legislation and the institutional requirements.

## Author Contributions

YH, HH, and YL conceived the research, drafted the manuscript, and performed the statistical analysis. DL and LW revised the manuscript and designed this study. All authors have read and approved the final manuscript.

## Conflict of Interest

The authors declare that the research was conducted in the absence of any commercial or financial relationships that could be construed as a potential conflict of interest.

## Publisher’s Note

All claims expressed in this article are solely those of the authors and do not necessarily represent those of their affiliated organizations, or those of the publisher, the editors and the reviewers. Any product that may be evaluated in this article, or claim that may be made by its manufacturer, is not guaranteed or endorsed by the publisher.

## Footnotes

1
www.Datadryad.org

2
http://www.R-project.org

3
http://www.empowerstats.com

## Abbreviations

CHF: congestive heart failure
IPTW: inverse probability of treatment-weighted
SMR: standardised mortality ratio
HF: heart failure
PSM: propensity score matching
RCT: randomised controlled trials
CVA: previous cerebrovascular accidents
IHD: ischaemic heart disease
DMI: diabetes mellitus on insulin
ASA-PS: American Society of Anaesthesiologists Physical Status
RDW: red cell distribution width
HR: hazard ratios
SD: standardised differences
CAD: coronary artery disease.
