# Supplementary material for: The Association Between Congestive Heart Failure and One-Year Mortality After Surgery in Singaporean Adults: A Secondary Retrospective Cohort Study Using Propensity-Score Matching, Propensity Adjustment, and Propensity-Based Weighting
Source: Front Cardiovasc Med. 2022 Jun 17;9:858068. doi: 10.3389/fcvm.2022.858068 (PMC9247191; doi:10.3389/fcvm.2022.858068)
Supplement: Supplementary file 2 [file Data_Sheet_2.docx]

**The association between** **congestive heart failure and** **one-year mortality after surgery in Singaporean adults: a secondary retrospective cohort study using propensity-score matching, propensity adjustment, and Propensity-based Weighting.**

**Running title:** Congestive heart failure and one-year mortality after surgery

Yong Han^1#^, Haofei Hu^2#^,Yufei Liu^3#^, Qiming LI^1^, Zhiqiang Huang^1^,Zhibin Wang^1^, Dehong Liu^1*^,Longning Wei^4^*,

^1^ Department of emergency, Shenzhen Second People's Hospital, Shenzhen 518035, Guangdong Province, China

^2^ Department of nephrology, Shenzhen Second People's Hospital, Shenzhen 518035, Guangdong Province, China

^3^Department of neurosurgery, Shenzhen Second People's Hospital, Shenzhen 518035, Guangdong Province, China

^4^ Department of emergency, Hechi People's Hospital, Hechi 547000, Guangxi Zhuang Autonomous Region, China

Yong Han^1#^, Haofei Hu^2#^,Yufei Liu^3#^ have contributed equally to this work.

*Corresponding author

Dehong Liu

Department of emergency, Shenzhen Second People's Hospital

No.3002 Sungang Road, Futian District,

Shenzhen 518035,

Guangdong Province,

China

E-mail: dhliu_emergency@163.com

*Corresponding author

Longning Wei^4^*

Department of emergency, Hechi People's Hospital,

No. 455 Jincheng Middle Road, Jinchengjiang District,

Hechi 547000,

Guangxi Zhuang Autonomous Region,

China.

E-mail: hong98712@163.com

**Table S1**  Missing variables

| Variables | Non-missing | missing |
| --- | --- | --- |
| ASA-PS | 68835 | 197(0.29%) |
| CVA | 66853 | 2179(3.16%) |
| IHD | 66602 | 2430(3.52%) |
| DMI | 68260 | 772(1.05%) |
| Creatinine Category | 68170 | 862(1.25%) |
| Anemia | 66556 | 2476(3.59%) |
| Stage of CKD | 61181 | 7851(11.37%) |
| Anesthesia | 69032 | 0 |
| Priority of Surgery | 69032 | 0 |
| Surgery Risk | 65597 | 3435(4.98%) |
| Race | 69019 | 13(0.02%) |
| Age | 69032 | 0 |
| RDW category | 63733 | 5299(7.68%) |
| Sex | 69032 | 0 |
| total | 940934 | 25514(3.70%) |

SD Standardized differences, CVA history of previous cerebrovascular accidents, IHD history of ischemic heart disease; DMI history of diabetes mellitus on insulin, CHF congestive heart failure; ASA-PS American Society of Anesthesiologists Physical Status. CKD chronic kidney disease; RDW red cell distribution width

**Table S2**  Relationship congestive heart failure and one-year mortality after surgery in different models of the original cohort before and after multiple imputations

| Variable | crude (HR 95% CI ) | Model I (HR 95% CI P) | model II (HR 95% CI P) |
| --- | --- | --- | --- |
| Before multiple imputations | |  |  |
| Non-CHF | ref | ref | ref |
| CHF | 5.645 (4.919, 6.477) <0.00001 | 3.478 (3.025, 3.999) <0.00001 | 1.402 (1.176, 1.672) 0.00017 |
| After multiple imputations | |  |  |
| Non-CHF | ref | ref | ref |
| CHF | 5.645 (4.919, 6.477) <0.00001 | 3.471 (3.019, 3.991) <0.00001 | 1.391 (1.198, 1.614) 0.00001 |

Crude model: we did not adjust other covariates

Model I: we adjust age, sex, race

Model II: we adjustsex, age, race, history of previous cerebrovascular accidents, history of ischemic heart disease, diabetes mellitus on insulin, priority of Surgery, surgical risk classification, American society of anesthesiologists physical status, grade of kidney disease, degree of anemia, type of anesthesia.

HR, hazard ratio; CI, conﬁdence interval; CHF: congestive heart failure

**Table S3** According to the percentile of the propensity score of the entire study population, the proportion of postoperative one-year mortality in patients with and without CHF.

|  | CHF | | | |  | Non-CHF | | | | Empirical OR* |
| --- | --- | --- | --- | --- | --- | --- | --- | --- | --- | --- |
| Percentile |  | No. | mortality | |  |  |  | mortality | |  |
|  | Score^*^ |  | No. | % |  | Score^*^ | No. | No. | % |  |
| 0 to 10 | 0 | 0 | 0 | 0 |  | 0.000 | 6608 | 7 | 0.106% |  |
| 10< to 20 | 0 | 0 | 0 | 0 |  | 0.001 | 7223 | 18 | 0.249% |  |
| 20< to 30 | 0.001 | 4 | 0 | 0 |  | 0.002 | 6883 | 64 | 0.930% |  |
| 30< to 40 | 0.003 | 3 | 1 | 33.333% |  | 0.003 | 6953 | 63 | 0.906% |  |
| 40< to 50 | 0.003 | 3 | 0 | 0 |  | 0.003 | 6850 | 77 | 1.124% |  |
| 50< to 60 | 0.004 | 13 | 0 | 0 |  | 0.004 | 7027 | 111 | 1.580% |  |
| 60< to 70 | 0.005 | 19 | 0 | 0 |  | 0.005 | 6732 | 144 | 2.139% |  |
| 70< to 80 | 0.011 | 53 | 2 | 3.744% |  | 0.009 | 6843 | 312 | 4.559% | 0.82 |
| 80< to 90 | 0.029 | 278 | 21 | 7.554% |  | 0.027 | 6634 | 625 | 9.421% | 0.80 |
| 90< to 95 | 0.082 | 315 | 35 | 11.111% |  | 0.080 | 3137 | 258 | 8.224% | 1.40 |
| 95< to 99 | 0.176 | 487 | 100 | 20.534% |  | 0.166 | 2274 | 295 | 12.973% | 1.73 |
| 99< to 100 | 0.463 | 236 | 66 | 27.996% |  | 0.430 | 455 | 108 | 23.736% | 1.28 |
| All patients | 0.115 | 1413 | 225 | 15.924% |  | 0.003 | 67619 | 2082 | 3.079% | 5.96 |

CHF: congestive heart failure; Empirical OR: Propensity-stratum-speciﬁc-treatment–mortality odds ratio; Score*: mean propensity score in percentile.

**Table S4 One-year mortality after surgery before and after propensity-score matching**

| Variable | Participants(n) | Death event(n) | Cumulative mortality (95% CI %) |
| --- | --- | --- | --- |
|  | Before matching | |  |
| total | 69032 | 2307 | 3.3 (3.2-3.5) |
| CHF | 1413 | 225 | 15.9(14.0-17.8) |
| Non -CHF | 67619 | 2082 | 3.1(2.9-3.2) |
|  | After matching | |  |
| total | 2630 | 326 | 12.4(11.1-13.7) |
| CHF | 1315 | 194 | 14.8(12.8-16.7) |
| Non-CHF | 1315 | 132 | 10.0(8.4-11.7) |

CI Confidence interval, CHF congestive heart failure

**Table S5 Associations between CHF and one-year mortality after surgery in the crude analysis, multivariable analysis, and four propensity-score methods analyses (restriction to participants with propensity score ≥ 0.05)**

| Cox proportional-hazards regression model | Adjusted variables | No. | HR | | 95%CI | P |
| --- | --- | --- | --- | --- | --- | --- |
| Crude |  | 6882 | 1.81 | 1.55, 2.12 | | <0.001 |
| Multivariable-adjusted model | Multivariable† | 6882 | 1.59 | 1.35, 1.88 | | <0.001 |
| Propensity score adjustment | Propensity score + Multivariable† | 6882 | 1.54 | 1.30, 1.82 | | <0.001 |
| Propensity score matching | Multivariable† | 1877 | 1.55 | 1.19, 2.02 | | 0.001 |
| IPTW | Multivariable† | 6882 | 1.49 | 1.36, 1.67 | | <0.001 |
| SMR-weighted | Multivariable† | 6882 | 1.45 | 1.18, 1.78 | | <0.001 |

HR, hazard ratio; CI, conﬁdence interval; IPTW, inverse-probability-of-treatment weighted; SMR, standardized mortality ratio.

Multivariable†: Adjusted for sexr, age, race, history of previous cerebrovascular accidents, history of ischemic heart disease, diabetes mellitus on insulin, priority of surgery, surgical risk classification,American society of anesthesiologists physical status, stage of CKD,, degree of anemia, type of Anesthesia.

**Table S6** **Associations between CHF and one-year postoperative mortality in crude analysis, multivariate analysis, and four propensity score methods (all participants after multiple imputation estimates of CHF information missing)**

| Cox proportional-hazards regression model | Adjusted variables | No. | HR | | 95%CI | P value |
| --- | --- | --- | --- | --- | --- | --- |
| Crude |  | 97443 | 5.10 | 4.54, 5.71 | | <0.001 |
| Multivariable-adjusted model | Multivariable† | 97443 | 1.24 | 1.01, 1.42 | | <0.001 |
| Propensity score adjustment | Propensity score + Multivariable† | 97443 | 1.26 | 1.11, 1.42 | | <0.001 |
| Propensity score matching | multivariable† | 4166 | 1.31 | 1.13, 1.78 | | 0.003 |
| IPTW | Multivariable† | 97443 | 1.16 | 1.11, 1.22 | | <0.001 |
| SMR–weighted | multivariate† | 97443 | 1.31 | 1.11, 1.54 | | 0.001 |

HR, hazard ratio; CI, conﬁdence interval; IPTW, inverse-probability-of-treatment weighted; SMR, standardized mortality ratio.

Multivariable†: Adjusted for sex, age, race, history of previous cerebrovascular accidents, history of ischemic heart disease, diabetes mellitus on insulin, priority of surgery, surgical risk classification ,American Society of Anesthesiologists Physical Status, stage of CKD,, degree of anemia, type of Anesthesia.

**Table S7** Effect size of CHF on one-year mortality in prespecified and exploratory subgroups

| Characteristic | No of participants | HR (95%CI) | P value | P interaction |
| --- | --- | --- | --- | --- |
| Age(years) |  |  |  | 0.2606 |
| 18 to <70 | 1666 | 1.687 (1.177, 2.418) | 0.0044 |  |
| ≥70 | 92 | 2.063 (0.241, 17.619) | 0.5083 |  |
| Stage of CKD | |  |  | 0.6143 |
| 1-2 | 990 | 1.829 (1.070, 3.127) | 0.0273 |  |
| 3-5 | 724 | 2.744 (1.642, 4.583) | 0.0001 |  |

Note 1:Above model adjusted for sex, age, race, history of previous cerebrovascular accidents, history of ischemic heart disease, diabetes mellitus on insulin, Priority of Surgery, surgical risk classification, American Society of Anesthesiologists Physical Status, Stage of CKD, Degree of anemia, Type of Anesthesia.

Note 2:In each case; the model is not adjusted for the stratification variable

HR, hazard ratio; CI, conﬁdence interval;

**Table S8** Univariate Analysis Between Variables and one-year mortality after surgery in origin cohort

| Variable | Statistics | HR 95% CI P |
| --- | --- | --- |
| ASA-PS |  |  |
| 1 | 16220 (23.496%) | Ref. |
| 2 | 38315 (55.503%) | 7.186 (5.202, 9.926) <0.00001 |
| 3 | 13145 (19.042%) | 42.703 (31.053, 58.724) <0.00001 |
| 4 | 1325 (1.919%) | 118.622 (85.053, 165.440) <0.00001 |
| 5 | 27 (0.039%) | 211.196 (105.428, 423.072) <0.00001 |
| CVA |  |  |
| No | 67155 (97.281%) | Ref. |
| Yes | 1877 (2.719%) | 3.560 (3.081, 4.115) <0.00001 |
| IHD |  |  |
| No | 62065 (89.908%) | Ref. |
| Yes | 6967 (10.092%) | 3.307 (3.014, 3.628) <0.00001 |
| CHF |  |  |
| No | 67619 (97.953%) | Ref. |
| Yes | 1413 (2.047%) | 5.645 (4.919, 6.477) <0.00001 |
| DMI |  |  |
| No | 66719 (96.649%) | Ref. |
| Yes | 2313 (3.351%) | 3.477 (3.041, 3.975) <0.00001 |
| Creatinine Category |  |  |
| Normal | 66884 (96.888%) | Ref. |
| High | 2148 (3.112%) | 7.120 (6.391, 7.933) <0.00001 |
| Degree of anemia |  |  |
| Normal | 50146 (72.642%) | Ref. |
| Mild | 10467 (15.163%) | 3.884 (3.478, 4.338) <0.00001 |
| Moderate | 8142 (11.795%) | 8.865 (8.046, 9.768) <0.00001 |
| Server | 277 (0.401%) | 20.364 (15.878, 26.117) <0.00001 |
| Stage of CKD |  |  |
| 1 | 41507 (60.127%) | Ref. |
| 2 | 20240 (29.320%) | 1.136 (1.022, 1.263) 0.01801 |
| 3 | 4674 (6.771%) | 3.345 (2.962, 3.779) <0.00001 |
| 4 and 5 | 2611 (3.782%) | 8.171 (7.309, 9.136) <0.00001 |
| Type of Anesthesia |  |  |
| General | 59133 (85.660%) | Ref. |
| Regional | 9899 (14.340%) | 1.192 (1.068, 1.330) 0.00172 |
| Priority of Surgery |  |  |
| Elective | 57007 (82.581%) | Ref. |
| Emergency | 12025 (17.419%) | 2.637 (2.421, 2.872) <0.00001 |
| Surgical risk classification |  |  |
| Low | 34468 (49.930%) | Ref. |
| Moderate | 30504 (44.188%) | 1.800 (1.641, 1.975) <0.00001 |
| High | 4060 (5.881%) | 5.144 (4.564, 5.799) <0.00001 |
| Race |  |  |
| Chinese | 49616 (71.874%) | Ref. |
| Malay | 6868 (9.949%) | 1.077 (0.946, 1.225) 0.26241 |
| India | 6016 (8.715%) | 0.788 (0.673, 0.922) 0.00300 |
| Others | 6532 (9.462%) | 0.415 (0.339, 0.509) <0.00001 |
| Age（year） |  |  |
| 18-29 | 7288 (10.557%) | Ref. |
| 30-49 | 19174 (27.776%) | 2.022 (1.416, 2.887) 0.00011 |
| 50-69 | 30269 (43.848%) | 7.574 (5.435, 10.555) <0.00001 |
| ≥70 | 12301 (17.819%) | 16.524 (11.848, 23.047) <0.00001 |
| RDW category |  |  |
| ≤15.7% | 62331 (90.293%) | Ref. |
| >15.7% | 6701 (9.707%) | 4.441 (4.067, 4.850) <0.00001 |
| Gender |  |  |
| Female | 35676 (51.680%) | Ref. |
| Male | 33356 (48.320%) | 1.435 (1.321, 1.558) <0.00001 |

HR, hazard ratio; CI, conﬁdence interval.

CVA history of previous cerebrovascular accidents, IHD history of ischemic heart disease; DM history of diabetes mellitus) on insulin, CHF congestive heart failure; ASA-PS American Society of Anesthesiologists Physical Status. CKD chronic kidney disease; RDW red cell distribution width.

**Table S9** Baseline characteristics of included participants and excluded participants with missing CHF information

|  | included participants | excluded participants | SD(%)(95% CI) |
| --- | --- | --- | --- |
| participants | 69032 | 28411 |  |
| Age(years) |  |  | 15.0 (14, 17) |
| 18 to <30 | 7288 (10.56%) | 3975 (13.99%) |  |
| 30 to<50 | 19174 (27.78%) | 8932 (31.44%) |  |
| 50 to <70 | 30269 (43.85%) | 11206 (39.44%) |  |
| ≥70 | 12301 (17.82%) | 4298 (15.13%) |  |
| Sex |  |  | 2.0 (0.01, 0.03) |
| Female | 35676 (51.68%) | 14956 (52.64%) |  |
| Male | 33356 (48.32%) | 13455 (47.36%) |  |
| Race |  |  | 4.0 (3.0, 6.0) |
| Chinese | 49609 (71.88%) | 19876 (69.97%) |  |
| Malay | 6865 (9.95%) | 2961 (10.42%) |  |
| Indian | 6015 (8.71%) | 2606 (9.17%) |  |
| Others | 6530 (9.46%) | 2964 (10.43%) |  |
| ASA-PS |  |  | 11 (10, 13) |
| 1 | 16168 (23.49%) | 5979 (25.43%) |  |
| 2 | 38211 (55.51%) | 12041 (51.22%) |  |
| 3 | 13105 (19.04%) | 4688 (19.94%) |  |
| 4 | 1324 (1.92%) | 788 (3.35%) |  |
| 5 | 27 (0.04%) | 11 (0.05%) |  |
| CVA |  |  | 52 (26, 79) |
| No | 65052 (97.31%) | 45 (81.82%) |  |
| Yes | 1801 (2.69%) | 10 (18.18%) |  |
| IHD |  |  | 37 (13, 62) |
| No | 59905 (89.94%) | 48 (76.19%) |  |
| Yes | 6697 (10.06%) | 15 (23.81%) |  |
| DMI |  |  | 101 (79, 123) |
| No | 65964 (96.64%) | 48 (59.26%) |  |
| Yes | 2296 (3.36%) | 33 (40.74%) |  |
| Creatinine Category |  |  | 0 (-2, 2) |
| Normal | 66072 (96.92%) | 11793 (96.95%) |  |
| High | 2098 (3.08%) | 371 (3.05%) |  |
| Anemia |  |  | 4 (2, 5) |
| Normal | 48162 (72.36%) | 18937 (71.92%) |  |
| Mild | 10156 (15.26%) | 4033 (15.32%) |  |
| Moderate | 7964 (11.97%) | 3182 (12.09%) |  |
| Severe | 274 (0.41%) | 177 (0.67%) |  |
| Stage of CKD |  |  | 1 (0, 3) |
| 1 | 36064 (58.95%) | 14212 (59.06%) |  |
| 2 | 18464 (30.18%) | 7176 (29.82%) |  |
| 3 | 4185 (6.84%) | 1665 (6.92%) |  |
| 4-5 | 2468 (4.03%) | 1009 (4.19%) |  |
| Anesthesia |  |  | 4 (2, 5) |
| General | 59133 (85.66%) | 23967 (84.36%) |  |
| Regional | 9899 (14.34%) | 4444 (15.64%) |  |
| Priority of surgery |  |  | 20 (18, 21) |
| Elective | 57007 (82.58%) | 21154 (74.46%) |  |
| Emergency | 12025 (17.42%) | 7257 (25.54%) |  |
| Surgery risk |  |  | 11 (10, 13) |
| Low | 33064 (50.40%) | 14938 (55.83%) |  |
| Moderate | 29053 (44.29%) | 10671 (39.88%) |  |
| High | 3480 (5.31%) | 1146 (4.28%) |  |
| RDW category |  |  | 1 (0, 3) |
| ≤15.7% | 57406 (90.07%) | 23289 (89.72%) |  |
| >15.7% | 6327 (9.93%) | 2667 (10.28%) |  |
| One-year mortality |  |  | 4 (2, 5) |
| No | 66725 (96.66%) | 27262 (95.96%) |  |
| Yes | 2307 (3.34%) | 1149 (4.04%) |  |

Values were n (%) or mean ± SD,

CVA history of previous cerebrovascular accidents,, IHD history of ischemic heart disease; DMI history of diabetes mellitus on insulin; CHF congestive heart failure; ASA-PS American Society of Anesthesiologists Physical Status; CKD chronic kidney disease; RDW red cell distribution width. SD Standardized differences, CI, conﬁdence interval;

**Fig.S1** **Comparison of propensity scores between CHF group and non-CHF group** **in original cohort**

Figure S1 showed The mean propensity score of participants with CHF was 0.017 (standard deviation, 0.049) compared with 0.163 (standard deviation,0.159) for participants without CHF(P<0.01) **in original cohort**

**Fig.S2 Comparison of propensity scores between CHF group and non-CHF group in matched cohort**

Figure S2 showed that in the matched population, the mean propensity score was 0.166 (standard deviation, 0.125) for both participants in the CHF and non-CHF groups, which was not statistically different（P=0.995）

Fig.S3 **Distribution of the propensity score for CHF and non-CHF participants are summarized in original cohort and in matched cohort.**

Figure S3a showed the distribution of the propensity score of for participants with congestive heart failure and without congestive heart failure in original cohort. There was a large difference in distribution between the two groups.

Figure S3b showed the distribution of the propensity score of for participants with congestive heart failure and without congestive heart failure in matched cohort. The distribution of CHF group and non-CHF group basically matched.
