# Supplementary figures and images for: The Association Between Congestive Heart Failure and One-Year Mortality After Surgery in Singaporean Adults: A Secondary Retrospective Cohort Study Using Propensity-Score Matching, Propensity Adjustment, and Propensity-Based Weighting
Source: Front Cardiovasc Med. 2022 Jun 17;9:858068. doi: 10.3389/fcvm.2022.858068 (PMC9247191; doi:10.3389/fcvm.2022.858068)

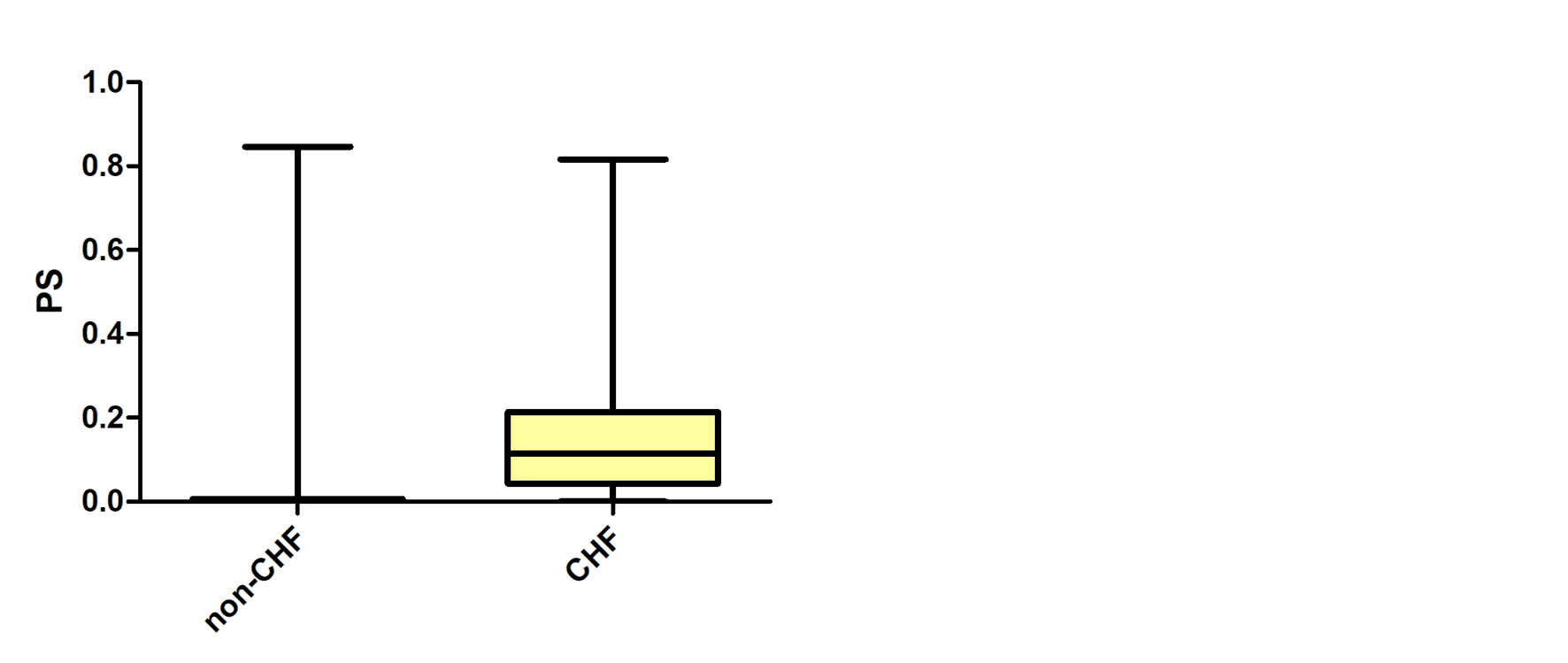

Supplement: Supplementary file 3 [file Image_1.TIF]

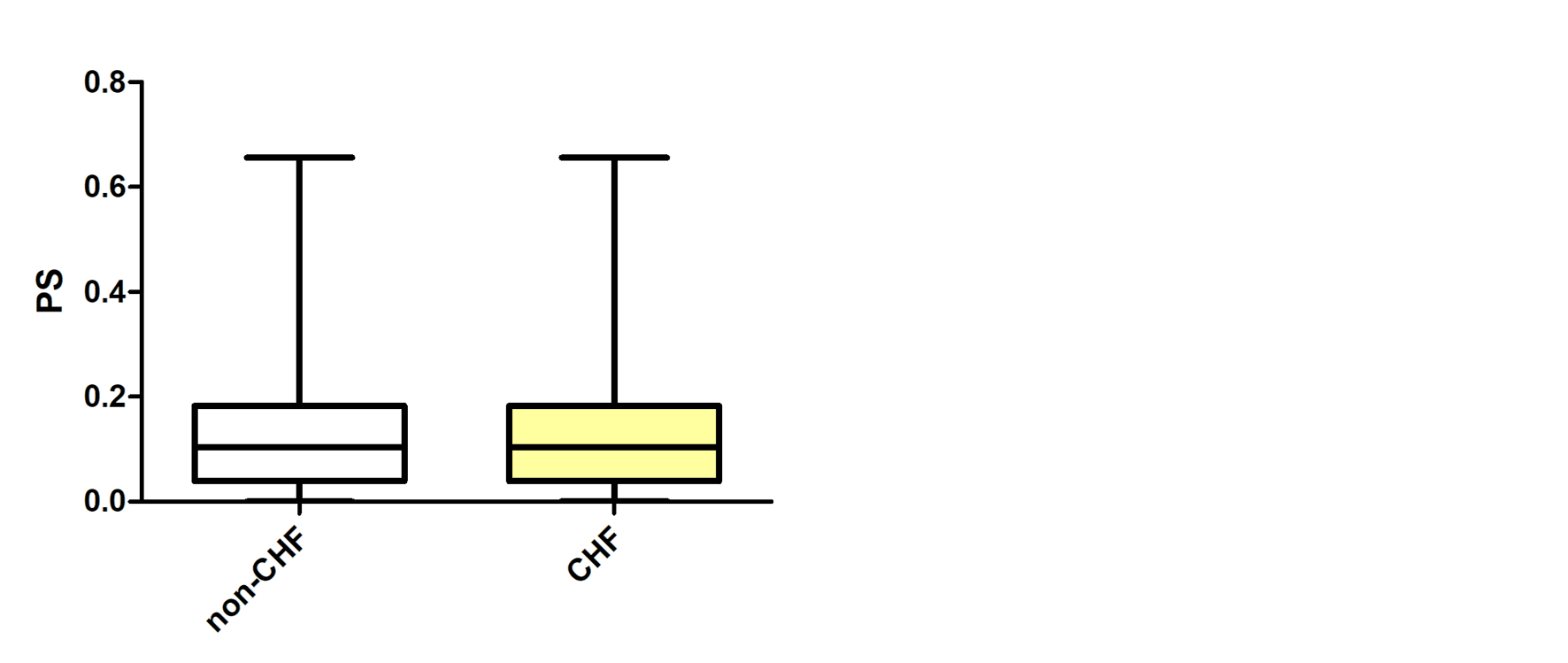

Supplement: Supplementary file 4 [file Image_2.TIF]

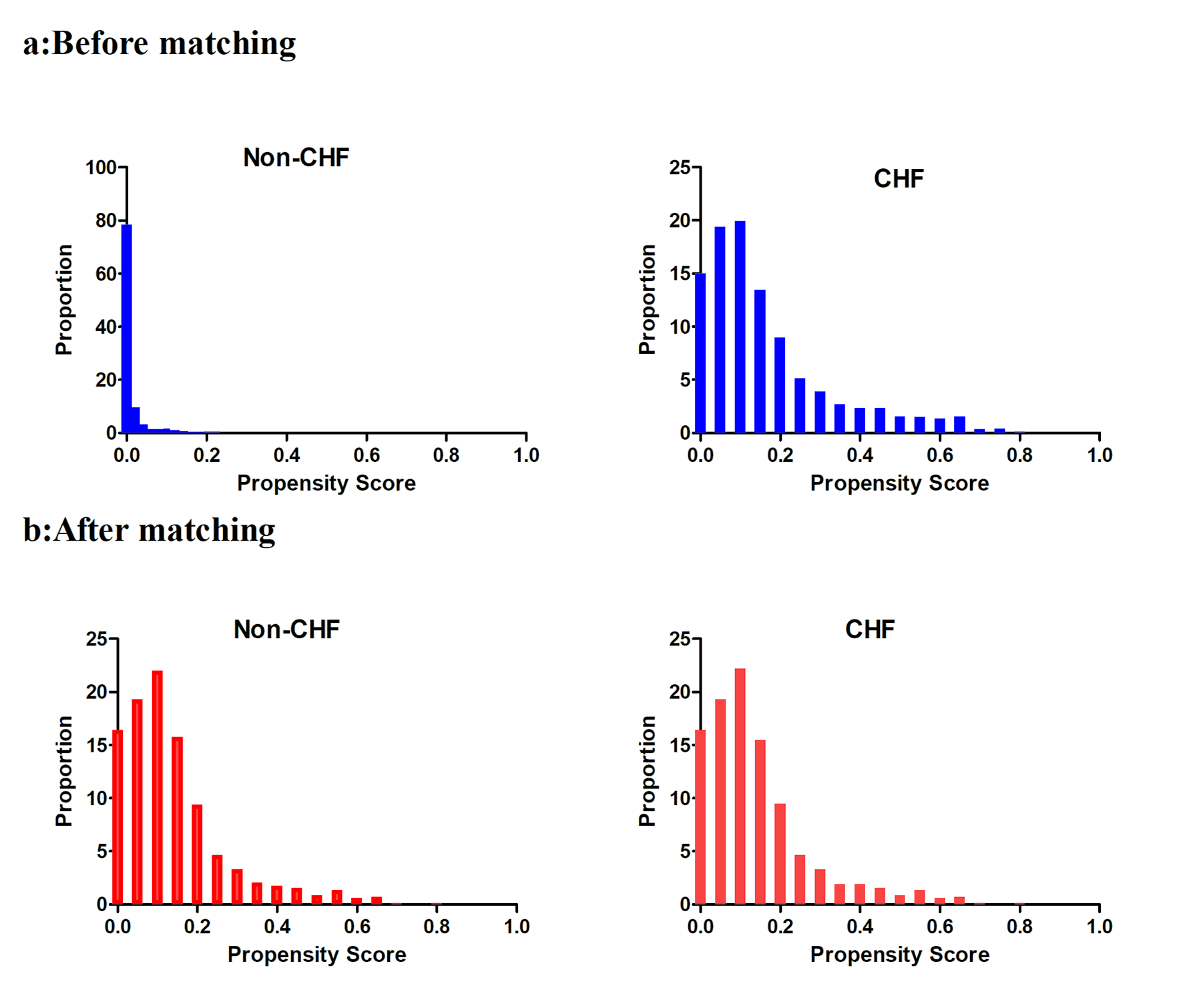

Supplement: Supplementary file 5 [file Image_3.TIF]
